# Using Intervention Mapping to Develop a Digital Self-Management Program for People With Type 2 Diabetes: Tutorial on MyDESMOND

**DOI:** 10.2196/17316

**Published:** 2020-05-11

**Authors:** Michelle Hadjiconstantinou, Sally Schreder, Christopher Brough, Alison Northern, Bernie Stribling, Kamlesh Khunti, Melanie J Davies

**Affiliations:** 1 Diabetes Research Centre University of Leicester Leicester United Kingdom; 2 Leicester Diabetes Centre NHS Trust University Hospitals of Leicester Leicester United Kingdom

**Keywords:** diabetes mellitus, type 2, technology, self-management

## Abstract

Digital health interventions (DHIs) are increasingly becoming integrated into diabetes self-management to improve behavior. Despite DHIs becoming available to people with chronic conditions, the development strategies and processes undertaken are often not well described. With theoretical frameworks available in current literature, it is vital that DHIs follow a shared language and communicate a robust development process in a comprehensive way. This paper aims to bring a unique perspective to digital development, as it describes the systematic process of developing a digital self-management program for people with type 2 diabetes, MyDESMOND. We provide a step-by-step guide, based on the intervention mapping (IM) framework to illustrate the process of adapting an existing face-to-face self-management program (diabetes education and self- management for ongoing and newly diagnosed, DESMOND) and translating it to a digital platform (MyDESMOND). Overall, this paper describes the 4 IM steps that were followed to develop MyDESMOND—step 1 to establish a planning group and a patient and public involvement group to describe the context of the intervention and program goals, step 2 to identify objectives and determinants at early design stages to maintain a focus on the strategies adopted, step 3 to generate the program components underpinned by appropriate psychological theories and models, and step 4 to develop the program content and describe the iterative process of refining the content and format of the digital program for implementation. This paper concludes with a number of key learnings collated throughout our development process, which we hope other researchers may find useful when developing DHIs for chronic conditions.

## Introduction

### Background

Type 2 diabetes (T2D) is a progressive lifelong condition affecting over 422 million people worldwide [1] and is associated with a number of severe macrovascular complications (damage to large blood vessels leading to coronary heart disease and stroke) and microvascular complications (damage to small blood vessels leading to kidney disease, blindness, and amputation). Diabetes self-management has been shown to reduce the risk of these related complications, improve one’s well-being, and reduce the UK National Health Service’s (NHS) health costs [2].

Self-management education can be delivered in several forms; one of these forms is via structured education programs. Structured education programs are nationally and internationally recommended for all people with T2D and meet nationally agreed criteria, including following an evidence-based curriculum that is theory-driven, evidence-based, and resource-effective, carrying out quality assurance of teaching standards and delivering regular audits [3]. Examples of such self-management education programs are diabetes education and self-management for ongoing and newly diagnosed (DESMOND) [4,5] and X-PERT (Diabetes education programme for people with T2D) [6] in the United Kingdom, Rethink Organization to iMprove Education and Outcomes [7] in Italy, and Structured Intensive Diabetes Education Program in South Korea [8]. These programs are available for people with T2D, both newly diagnosed and established [9].

In 2008, the National Institute of Health and Clinical Excellence published guidelines to recommend the availability of group-based structured education programs as a key priority for diabetes management [10]. Evidence has shown that group-based structured education programs are essential for effective diabetes management, aiming to improve people’s knowledge, skills, and confidence, enabling them to take control of their own condition. Effective education programs have also been shown to improve biomedical (weight and glucose levels), behavioral (ie, physical activity), and psychosocial outcomes (ie, diabetes-related distress and self-efficacy) [9]. Various barriers, however, appear to be associated with low uptake and attendance at structured education programs [11,12], with suboptimal access to programs commonly noted [13]. Attendance and accessibility for patients may also be compromised by health care professionals’ insufficient advocacy for structured education programs, as well as limited infrastructure and capacity to offer face-to-face programs [14].

Although referrals to structured education have increased over the last 3 years, attendance at such programs have decreased, and attendance rates are as low as they were in 2009 [15]. This is why a recent call by NHS England in the UK advised that people with diabetes are offered face-to-face programs, and those who cannot attend must, then, be offered a digital alternative [16].

Digital health interventions (DHIs, which are any digital-based intervention, such as smartphones, websites, text messaging) [17] have become a means of delivering care and offering support to facilitate behavior change in individuals with long-term conditions. Mounting evidence indicates that the use of digital technology for diabetes self-management is an effective way to support people to live well with their diabetes [18,19], with programs showing improvements mainly in glycemic control [18,20].

DHIs in other chronic conditions have been shown to be cost-effective, with a recent cost-effectiveness study depicting the benefits and cost-effectiveness of digital-based psychological interventions for reducing behavioral stress management and anxiety [21,22]. Information derived from DHIs can be kept up to date more easily with local and national guidelines and recommendations. DHIs are neither geographically nor time restrained. Indeed, barriers associated with face-to-face structured education programs, such as time and transport, are often easier to address with digital interventions, as users have the opportunity to access Web-based programs and read the material at their own time and pace.

Appropriate steps are essential when developing evidence- and theory-based digital programs to ensure they are implemented effectively in the real world [23]. Despite DHIs becoming more and more available to people with diabetes, the development strategies and processes undertaken can be somewhat unclear. With theoretical frameworks available in the current literature, such as Bartholomew’s intervention mapping (IM) framework [24], it is important that DHIs follow a shared language and communicate a robust development process appropriately and systematically [25].

### Intervention Mapping

**Figure 1:**
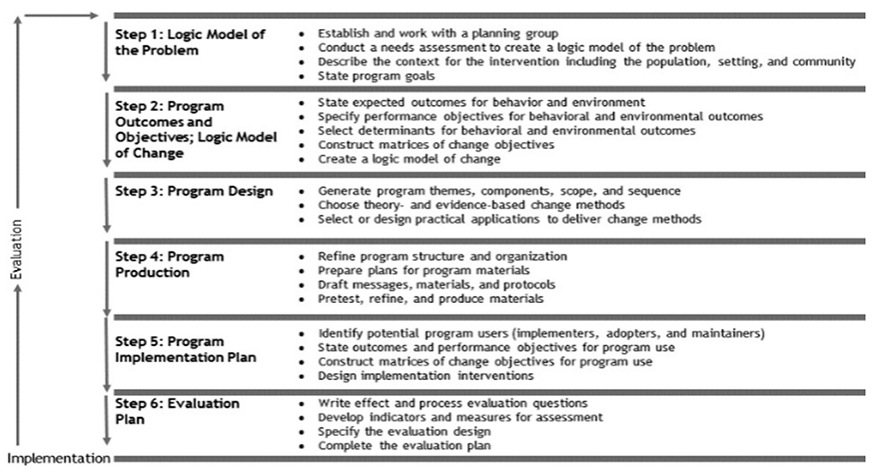
Intervention mapping framework, Bartholomew.

IM, a 6-step framework, is often applied to guide behavior change interventions and health education development. This evidence-based approach, although presented as a series of 6 steps, follows a more iterative process that bridges the gap between theory and practice. With this in mind, we aimed to develop our digital program based on a systematic and robust process such as IM, to ensure that our evidence-based program can be implemented in real-world settings. The development process for MyDESMOND was guided by the first 4 steps of IM, as illustrated in Figure 1: (1) conduct a needs assessment, (2) specify program outcomes and objectives, (3) design program and apply theory, and (4) refine program development. The last 2 steps (5) and (6) of the IM framework for MyDESMOND (adoption, implementation, and evaluation plan) are currently being evaluated.

### Aim

This paper describes the systematic developmental process that we undertook to adapt the content and evidence of an existing face-to-face structured self-management education program (DESMOND) for T2D and translate it into a digital self-management program (MyDESMOND). This process is presented based on the IM framework [24].

## Methods

### Step 1: Needs Assessment

#### Establish and Work With a Planning Group

A multidisciplinary team (psychologist, dietitian, diabetes specialist nurses, and experts in physical activity and digital health) was brought together to provide expertise in the psychosocial and clinical aspects of T2D, digital health, and behavior change research. Throughout the design process, our planning team worked alongside a Web-designer to ensure that the logistics and digital platform would be appropriate for a UK-based program.

Patient and public involvement was essential for our development process [26]. Throughout our process, we worked closely with people with T2D who provided feedback on the content: format and usability of MyDESMOND (see Step 4 Program Development for further details).

#### Conduct Mixed Methods Needs Assessment

We conducted a preliminary scoping search, and together with the findings of our existing research studies, we aimed to collate key learning points to explore and identify the key program goal and key behavioral determinants of MyDESMOND.

##### Scoping Literature

Self-management involves 3 key tasks (medical, role, and emotional) and consists of core self-management skills, including problem-solving, decision making, resource utilization, and action planning [27]. Digital self-management interventions over recent years have provided users with the knowledge and skills required to make a lifestyle change and improve their self-care. Enabling people with diabetes to better understand their conditions has been shown to improve coping strategies and reduce health-related anxiety and depression [18].

In addition to knowledge and improved coping skills, social support has also been linked as a major predictor of, and positive influence on diabetes self-management. A systematic review, which explored the role of social support on biomedical and psychosocial outcomes, as well as behavior change, reported a positive impact on the aforementioned outcomes [28]. In addition, recent diabetes literature has also illustrated a strong association between social support and improved diabetes self-management and self-efficacy [29].

A qualitative evaluation of patient and health care professional (HCP) views on barriers to self-management and multimorbidity (including diabetes) identified 3 main factors that influence self-management: (1) capacity (access and availability of resources, time, and knowledge), (2) responsibility (the degree to which patients and HCPs agree about self-management), and (3) motivation (willingness to take-up types of self-management practices) [30].

#### Systematic Reviews

We conducted focused synthesis of work in 2 areas: (1) a systematic review and meta-analysis to critically appraise and quantify the evidence on the effect of digital interventions that aim to improve well-being in people with T2D [31], and (2) a more recent systematic review of systematic reviews to explore and synthesize evidence around the applications of digital patient education in T2D and cardiovascular disease [32].

##### Systematic Review and Meta-Analysis

The systematic review and meta-analysis [31] identified preliminary evidence suggesting that digital interventions with significant well-being outcomes (in this case, depression or diabetes-related distress outcomes) shared common characteristics: (1) all interventions provided professional-led support, (2) interventions consisted of both asynchronous and synchronous communication, and (3) intervention duration was between 2 and 6 months. This review also identified that the common behavior change techniques (BCTs) adopted by effective digital interventions were *information provision* and *monitoring*.

The aim of this review was to examine if digital-based interventions improve well-being outcomes such as depression and diabetes-related distress; however, no significant improvements were found following a meta-analysis in depression (*P*=.15) or distress (*P*=.43). Nevertheless, the review did take into consideration that the number of studies included in the meta-analysis was low, and thus, conclusions on the meta-analysis were considered with caution.

On the basis of the findings above, we considered the following elements in our development work: (1) to have a professional-led, rather than peer-led program, (2) to include some form of *monitoring* element and feedback to users, and (3) to include both direct and indirect communication, in the form of a chat forum or messages.

##### Systematic Review of Systematic Reviews

This systematic review of the reviews [32] explored and synthesized the scope of digital patient education in T2D on patient outcomes. Our findings indicated that biological, behavioral, and psychological outcomes, as well as knowledge and self-efficacy for people with T2D, had shown examples of benefits from digital patient education. In addition, outcomes that most consistently showed improvement with the intervention (irrespective of effect size) were weight, physical activity, knowledge, social support, and quality of life. These findings suggest that digital patient education has a wide range of benefits for people with T2D. Therefore, we considered these findings in our development work and ensured that our program incorporated biological, behavioral, and psychological outcomes. Self-efficacy was also another crucial outcome, which we felt was important for behavior change (see additional information in theories, Step 3).

#### Our Current Face-to-Face Self-Management Program (DESMOND)

Our face-to-face self-management program [33] was developed for people with newly diagnosed T2D with the aim that participants would focus on lifestyle behaviors (such as food choices and physical activity), consider their personal risk factors, and choose a specific achievable goal to work on. This program is delivered for 6 hours in the community by 2 trained educators and is based on psychological theories of learning: Leventhal common sense theory [34], dual-process theory [35], and social learning theory [36], and the philosophy of the program was grounded on patient empowerment [37]. On the basis of the evaluation of DESMOND [33], this group self-management program showed greater improvements in weight loss and positive improvements in illness beliefs. Our aim was to adopt key elements of DESMOND that we considered crucial to the effectiveness of this program, including group dynamic and peer support, interactive activities, and setting a personal goal to help with behavior change. To ensure that elements were retained in the adapted format and remained suitable for a digital format, we found it was essential to revisit and underpin theories to our digital program that focused on the process of change. Thus, with the aforementioned key elements in mind, we aimed to provide a virtual environment whereby Web users would have the opportunity to share thoughts and experiences in the form of a chat forum and retain the peer support element. Interactive activities were developed to ensure that information was not provided in a didactic way, but instead was provided in a way whereby Web users could reflect on answers and aid their confidence. Similar to DESMOND, MyDESMOND users were able to assess which behavior change is important to them and tailor a personal action plan suitable to their environment and needs. Users would then be able to revisit their action plan to assess their progress. From preliminary feedback, participants expressed satisfaction with the content of the program (lifestyle behaviors) and reported they had gained knowledge and learned new skills in healthy living.

#### Our Digital-Based Self-Management Program

##### BabySteps

In 2017, we developed our first digital-based self-management program on a responsive digital platform to promote physical activity in women with a history of gestational diabetes (BabySteps) [38]. This digital program consisted of functions such as information material, step challenges, chat forums, and health trackers, and these functions were based on gamification and BCTs, including problem-solving, coping skills, and action planning. With the BabySteps trial coming to an end, we carried out feedback sessions with 12 participants to explore user experience and satisfaction. Preliminary findings have helped structure MyDESMOND in several ways: (1) DHIs are needed for chronic conditions, (2) to adopt a new behavior, people need to learn and practice self-care skills, (3) the navigation on apps plays a crucial role in digital uptake, (4) people engage with digital programs if it comes from a trustworthy source, and (5) peer support is essential in DHIs.

##### MyWellbeing

It was important to consider that BabySteps was a prevention program with a focus on physical activity (ie, increase steps and reduce sedentary behavior). We therefore supplemented our needs assessment step with further work conducted by our lead author [39] to develop a digital emotional support program to improve diabetes-related distress in T2D (MyWellbeing). This additional work aimed to complement the evidence behind MyDESMOND by incorporating the element of emotional management. This work adopted a person-centered approach [40] and gathered evidence from exploratory mixed methods research work (which will be published elsewhere). Most importantly, this work was based on ongoing patient and public involvement (PPI) engagement to support the development of this digital-based program. Key learnings from the PPI groups (which consisted of people with T2D and carers) were as follows: (1) incorporate links to useful external resources, (2) design a program with a positive language and motivational content, (3) incorporate *ask the expert* function, (4) use short videos to provide information, (5) include quizzes as a form of interactive learning, and (6) consider a *frequently asked question* page to enhance engagement.

#### Identify Program Goals

Overall, the outcomes of the needs assessment provided the evidence for our program goals and defined the purpose of our digital program, which was to provide a digital self-management program to people with T2D to improve medical, behavioral, and emotional self-management. This was developed with the aim to improve and maintain self-management behaviors, improve knowledge, promote self-care skills, increase self-efficacy, and improve well-being.

#### Create a Logic Model of the Problem

**Figure 2:**
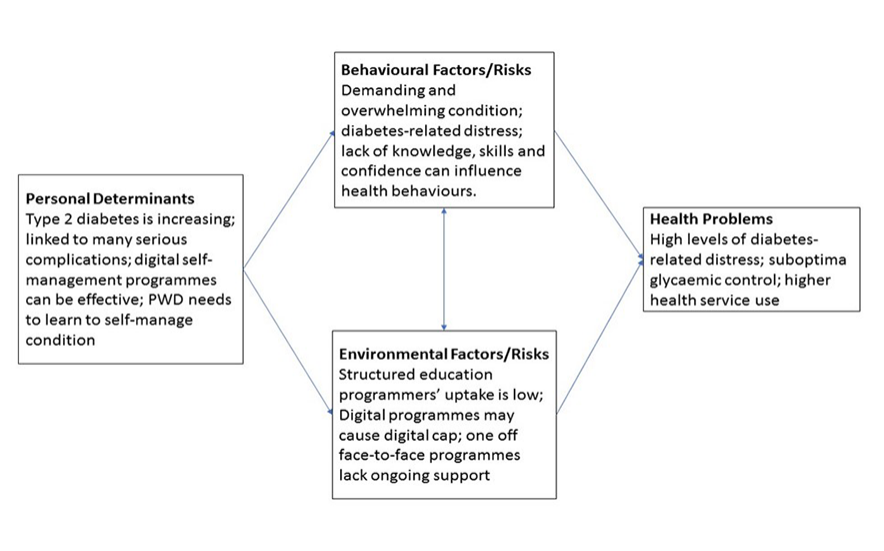
Logic model of the problem, step 1 IM.

On the basis of our scoping literature and mixed methods studies (as part of step 1), we produced a logic model of the problem, illustrating the link between behavioral determinants and the behavioral and environmental factors, which lead to the targeted health problems (Figure 2).

### Step 2: Behavioral Determinants and Performance Objectives

#### Select Behavioral Determinants and Performance Objectives

This step involved developing the specific change objectives and identifying specific behaviors that determine behavior change for this target population. Informed by the findings from our needs assessment, our planning group identified 5 key behavioral determinants and 5 performance objectives (objectives to be accomplished by the individual to achieve the program goals), which we anticipated would influence the behavioral outcome (Table 1). In detail, behavioral determinants were identified as follows: (1) knowledge, (2) self-efficacy, (3) social support, (4) intention, and (5) behavioral skills. Performance objectives were defined as follows: (1) learn about T2D, (2) be motivated to self-manage, (3) increase access to resource, (4) engage with the digital program, and (5) learn about self-care behaviors. The behavioral determinants listed in the matrix table (Table 1), derived from the Capability, Opportunity, Motivation, Behaviour (COM-B) model (which we explain in the following section), focus on factors that we identified as important in this target population. In this step, we elaborate on the strategies required to ensure that performance objectives are met, and subsequently, determinants are addressed (Table 1).

**Table 1:** Matrix of objectives.

Behavioral determinants	Performance objectives	Strategies
Knowledge	Learn about T2D^a^	Users gain knowledge; Information on healthy living, stress management, medication, physical activity, and diet
Self-efficacy	Be motivated to self-manage	Users formulate their own goal setting and action plan via patient testimonials and *expert* videos; Users are exposed to modeled behavior
Social support	Increase access to resource	Users become aware of local support; Users receive support not only from HCPs^b^ but also from peers
Intention	Engage with the digital program	Users engage with program functions tailored to their needs; intention to change behavior
Behavioral skills	Learn about self-care behaviors	Users practice self-care behaviors

^a^T2D: type 2 diabetes.

^b^HCP: health care practitioner.

### Step 3: Theory-Based Intervention Methods and Practical Applications

#### Identify Theory-Based Methods and Select Practical Applications

In a complex environment, dealing with influences on an individual, social, and cultural level, we aimed to adapt and develop a digital program that was theory-based to break down targeted health behaviors. Theory-based interventions are more effective than non–theory-based interventions and can provide a clear understanding of what works in terms of adapting interventions across populations, behaviors, or contexts [41]. Thus, we adapted the following theoretical frameworks and models to the development of our digital program.

##### Corbin and Strauss Model on Self-Management Framework

A key conceptual framework of self-management was developed by Corbin and Strauss. Self-management programs (face-to-face or digital) often include at least one of the three main overarching areas: cognitive (such as knowledge, understanding, and self-efficacy), behavioral (such as lifestyle changes and managing medication effectively), and emotional (such as distress, shame, and depression) [21]. On the basis of the Corbin and Strauss model, we aimed to adapt and develop an effective program that considers all three elements for self-management equally: cognitive, emotional, and behavioral [42].

##### Capability, Opportunity, Motivation, Behaviour Model and the Taxonomy of Behavior Change Techniques

**Figure 3:**
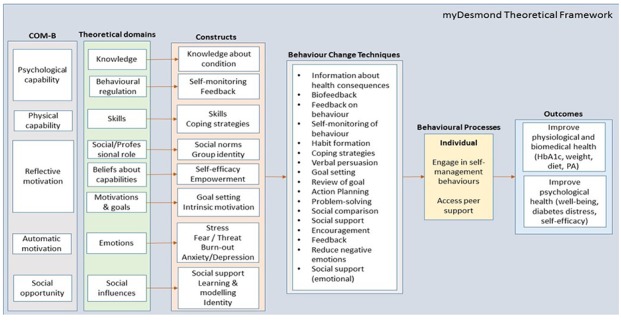
MyDESMOND theoretical framework.

We applied the COM-B model as it provides a common language for the reporting of behavior change interventions [43]. Atkins et al argued the importance of including behavioral determinants and BCTs in the development process of interventions, as these may influence and change health behavior [44,45]. Interventions targeting behavior determinants and behavior change have also been shown to be more effective [46]. Informed by Michie’s Behavior Change Wheel (a systematic guide to designing interventions) [43], we populated a theoretical framework for MyDESMOND to identify the relevant mechanisms of action, component constructs, and BCTs, all aimed to inform the individual and organizational processes and health outcomes (Figure 3). Our mechanisms of action, which are processes that influence behavior, included key constructs: knowledge (an awareness of the existence of a condition), behavioral regulation (self-efficacy and feedback), skills (an ability acquired through practice), beliefs about capabilities (belief and acceptance of one’s ability to perform), social and professional role and identity (one’s displayed behaviors and qualities within a social setting), emotions (one’s reaction and attempt to deal with a significant event), and social influences (interactions that influence one’s thoughts, feelings, emotions). Table 2 illustrates the relationship between the mechanisms of action, BCTs, and the practical application to implement change methods in MyDESMOND, an important factor for program development.

##### Health Action Process Approach Model

Despite the application of the behavior change taxonomy, it was still essential to adopt a model that helped explore the process of change and helped explain how individuals decide to engage in healthy behavior. Known as a *process* model, the Health Action Process Approach (HAPA) model informed the development of our digital program. Intention alone does not always change behavior [47], thus with the guidance of action planning and coping planning (both incorporated in our digital program), we aimed to build a program that focused on two processes: goal setting and goal pursuit. With self-efficacy as an additional HAPA construct, we know that people with higher self-efficacy are more likely to sustain the behavior; thus, we ensured that MyDESMOND also incorporated elements for this construct.

##### Social Support Theory

Social support plays an integral part in the management of diabetes and is often associated with health behaviors such as physical activity. Thus, it was deemed appropriate to base our digital program on a social support-focused theory. We considered all 4 types of social support [48]: *emotional support*, which focuses on sharing life experiences and reassurance; *instrumental support*, which can include practical support such as material or services that users can access directly; *informational support*, which involves evidence-based information or advice; and *appraisal support*, which includes concepts such as constructive feedback and affirmation. Lakey and Cohen’s [49] Social Support Theory was adopted to illustrate the relationship between *digital* social support and individuals’ appraisals and coping skills, which can, in turn, mediate distress levels and improve overall well-being and T2D self-care.

**Table 2:** Design of practical applications.

Mechanisms of action (accompanied by BCTs^a^)	Practical applications	Text description
Knowledge (information about health consequences, biofeedback, feedback on behavior)	Educational material; Interactive quiz; Animation videos; Decision tracker	Provide information about T2D^b^, complications, medication, and healthy lifestyle, including weight, diet, and physical activity; track and receive feedback on health.
Behavioral regulation (self-monitoring of behavior)	Sync steps; Leader board; Track daily targets	App users are encouraged to record dietary habits, weight, HbA_1c_^c^, well-being, blood glucose, cholesterol, and smoking habits, to sync and monitor steps, and complete an action plan for desired behavior.
Skills (coping strategies and habit formation)	Educational material; Interactive quiz; Chat forum	Provide information about a healthy lifestyle; encourage app users to record their tracks; opportunity to share strategies and experiences with other users.
Social and professional role (no BCTs linked to this domain)	Expert videos; Ask the expert	Highly respected experts provide (in the form of videos or emails) and present information and key messages about T2D, well-being, diet, physical activity, and other relevant information.
Beliefs about capabilities (verbal persuasion to boost self-efficacy)	Ask the expert; Chat forum; Patient testimonials	Verbal reassurance and encouragement among users that may boost their confidence and encourage them to continue with their action plan and behavior change
Motivations and goals (goal setting, review of goal, action planning, problem- solving)	Action Plan; Daily targets; Leader board; Personal achievements; Challenges	App users have access to functions and are encouraged to take part in challenges and set their daily targets. They are also encouraged to complete an action plan for target behaviors.
Emotions (reduce negative emotions, social support [emotional])	Chat forum; Whiteboard animation; Educational material; Decision tracker; Quiz	Provide information in the form of whiteboard animations, quizzes, and educational material around burnout, distress, depression, and well-being; use of a chat forum to provide an additional element of peer support.
Social influences (social comparison, social support, encouragement)	Patient testimonials; Chat forum; Leader board; Personal achievements; Challenges	App users have access to videos of peers discussing and sharing experiences of their journey. Chat forums are available for users who wish to share questions and thoughts—this platform acts as a mode for users to encourage one another.

^a^BCT: behavior change technique.

^b^T2D: type 2 diabetes.

^c^HbA_1c_: glycated hemoglobin.

### Step 4: Program Development

#### Develop Program Content

On the basis of key learnings from our face-to-face program (DESMOND) and our digital programs (BabySteps and myWellbeing) in combination with our theory-based approach, we had a clear idea of the program themes, material list, and aim of MyDESMOND. With the involvement of our multidisciplinary team, we designed scripts and material in line with the Corbin and Strauss self-management framework, incorporating content around medical, role, and emotional management. Once the content was adapted and developed, we liaised with our Web-designer and information technology team to map the material (ie, text, videos, and quiz) onto an appropriate digital platform that was easy to access from any electronic device (ie, desktop computer, smartphone, and tablet). MyDESMOND was developed to meet the objectives detailed in step 3. The following topics are included in MyDESMOND: *What is T2D, Medication, Complications of diabetes, Food choices, Physical Activity, Sedentary behavior, Emotions, Diabetes-related distress, Setting goals, and Relapse*.

#### Refine Content and Format

In addition to our previous work mentioned in the above sections, it was essential that we also involved patient and public feedback throughout the development of MyDESMOND. Furthermore, PPI work with early users helped inform and refine the content and format of our digital program. PPI work took place based on an iterative process, whereby 9 PPI members were provided logging details to *have a go* at MyDESMOND at various points of the development phase and provide feedback. Examples of PPI feedback and suggestions, which refined MyDESMOND included the following: (1) to provide the chat forum function as optional to the user (and not mandatory), as not all users would seek Web-based interaction with peers; (2) to include a separate section on well-being and discuss about diabetes-related distress and negative emotions in a nonjudgmental way—this suggestion from the PPI members reiterated issues around stigma and blame; (3) to provide weekly additional sessions on well-being, physical activity, and food choices—this change resulted in the development of additional mini videos to ensure that the users received regular information on important topics; and (4) to introduce mini videos of testimonials of MyDESMOND users sharing personal experience. Additional feedback received from our PPI members included (1) the navigation system of MyDESMOND was easy to use, (2) to make our logo clear to reassure that we are a reliable and trustworthy source; (3) digital ongoing support is much needed poststructured education programs; therefore, all content must be available at all times to the users.

#### MyDESMOND Platform

**Figure 4:**
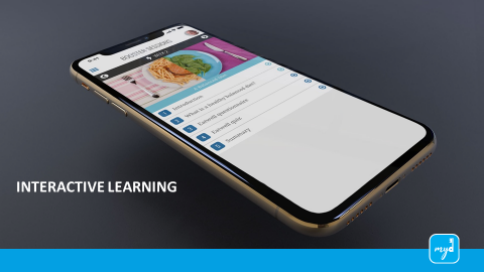
Users can learn about T2D and self-management activities and refresh knowledge with 8-weekly additional sessions (educational material, interactive quiz, animations) on MyDESMOND.

**Figure 5:**
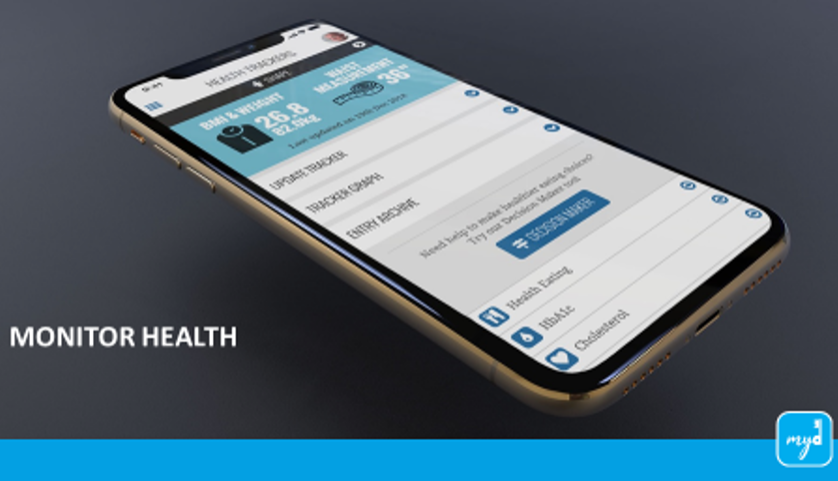
MyDESMOND can track weight, blood pressure, HbA_1c_, diet, and cholesterol.

**Figure 6:**
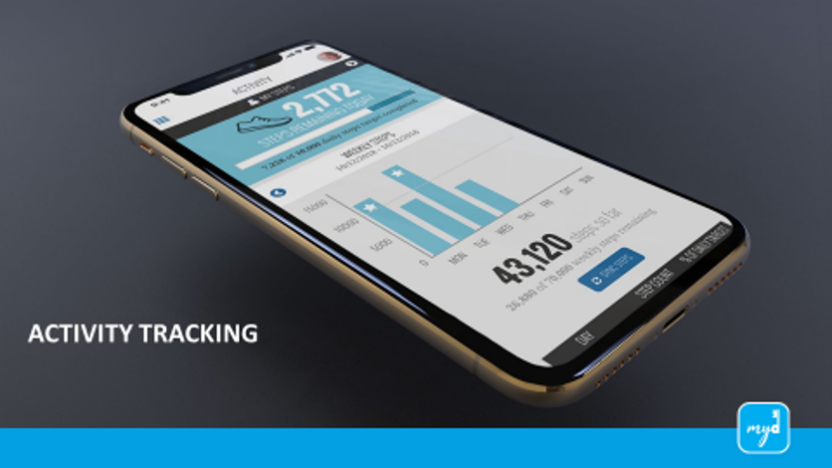
MyDESMOND can track activity levels and steps.

The interface and features were designed as such to improve diabetes self-management practices. The focus was directed toward daily step count, challenges, notifications, and the release of weekly education bite-sized sessions. These short sessions consisted of animations, gamification, quizzes, and games designed to be no more than 5 min in duration. Features include interactive learning, monitor health, activity tracking, ask the expert, community (share ideas, experiences, and questions with others in a chat forum), goal setting, challenges (complete challenges with other users on global leader boards to increase steps and focus on personal achievements), and invite a buddy (invite family and friends to join the program). Figures 4-6 present examples of features and description of MyDESMOND content.

**Figure 7:**
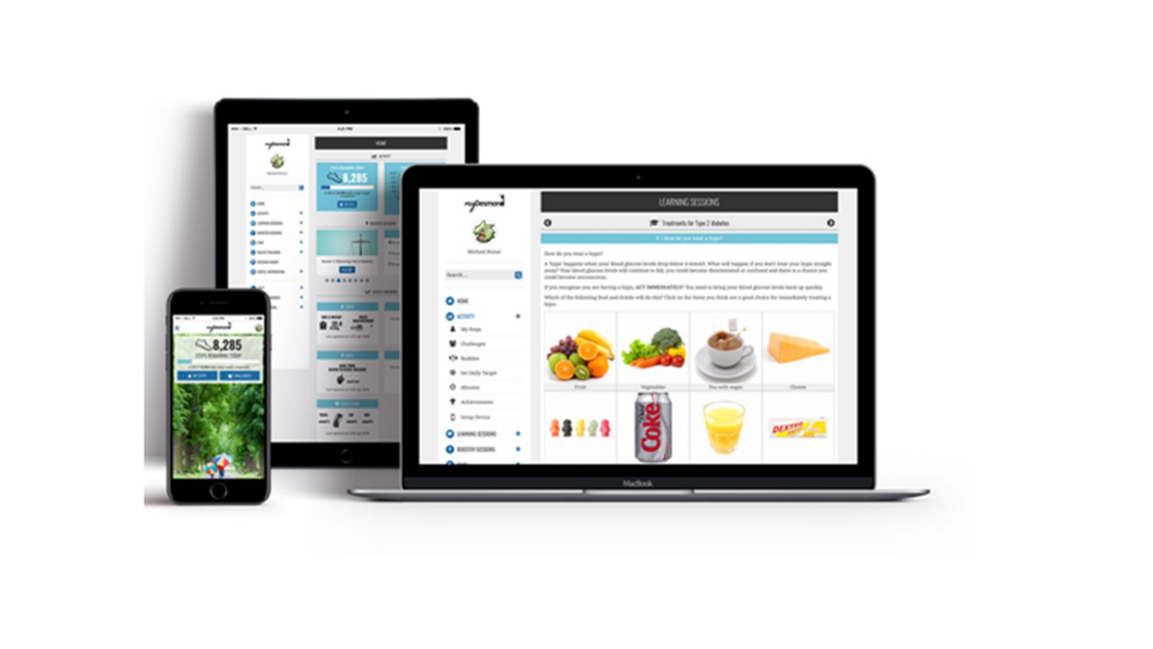
MyDESMOND operates like a native app.

MyDESMOND is accessible on any digital device (tablet, desktop computer, and smartphone). The platform was built using progressive Web applications (PWAs), a type of mobile app delivered through the Web. It operates similar to a native app, but its responsive design allows it to work across all modern browsers and devices (smartphone, tablet, and desktop computer; see Figure 7).

The system was developed using the Zend framework [50] and a MySQL database [51]. The app includes an application programming interface built with Zend that is used to manage data between the AngularJS PWA and the database. All identifiable user data, comments, and *Ask the expert* messages are encrypted and stored securely on the database. All data are stored on servers in the United Kingdom and are fully compliant with the latest industry standards for security and General Data Protection Regulations.

Both functional and nonfunctional testing was carried out throughout each step of the development phase by our research team, project-development team, Web-designers, PPI group, and external subject matter experts (SMEs). External SMEs carried out a number of security tests, including penetration testing (to ensure data remains safe) and a source code review (to ensure it complied with Open Web Application Security Project level 2). The purpose of this security testing was to attempt to identify any potential or actual vulnerability to an attack launched across a computer network that could threaten the confidentiality, availability, and integrity of the information being stored and processed.

## Discussion

### Summary

In this paper, we described the application of the IM framework for the adaptation and development of a digital self-management program, MyDESMOND. This paper brings a unique perspective to digital development, as it illustrated the process from adapting and translating the evidence and content of a face-to-face education program (DESMOND) to developing a digital version of it.

We described the 4 IM steps that were followed to develop MyDESMOND. In step 1 of the IM process, we established a planning group and a patient and public involvement group and described the context of the intervention and program goals. In step 2, we identified objectives and determinants at early design stages to maintain a focus on the strategies adopted. Step 3 focused on generating the program components underpinned by appropriate psychological theories and models. This led to step 4, which described the adaptation and development of the program content and the iterative process of refining the content and format of MyDESMOND.

### Strengths and Limitations

This paper has described in detail the three key perspectives of IM that informed the development of MyDESMOND: the use of evidence, the participation and engagement of stakeholders, and the use of theories and BCTs. Overall, our development work was based on a well-established and comprehensive theoretical framework, which has been used extensively in the development of behavior change programs.

The development process was heavily guided by stakeholder and PPI input, to ensure that we received regular feedback throughout the development phase and to ensure that the program was designed appropriately and was relevant to the target population, culturally and contextually. This approach helped refine and structure the format and content of MyDESMOND to reflect the unmet needs of this population accordingly. It is also important to highlight that although our needs assessment and evidence synthesis were predominantly based on findings from systematic reviews, our evidence was also based on rigorous research studies evaluating face-to-face and digital programs.

Some of the main limitations of IM, which we experienced during this development process, are the complexity and time-consuming aspects of this framework. Following a robust process such as IM, which included conducting systematic reviews, synthesizing evidence from existing studies, obtaining regular feedback from stakeholders, and identifying key theories and BCTs, required substantial time and resources from our team. Nevertheless, we found IM to be an extremely useful resource, which helped develop the best possible digital self-management program for our people with T2D.

### Lessons Learned

DHIs in diabetes self-management and other chronic conditions are increasing. It is important that future research recognizes the complexity of DHIs and continues to adopt a robust development process informed by evidence and theory. During the development of MyDESMOND, we identified key learning points that might be helpful to other researchers developing DHIs for chronic conditions.

### Multidisciplinary Team

The expertise of our research, project development, and clinical team members was critical to the successful development of the content, including diabetes education, diabetes clinical expertise, and behavior change. Together with experts in Web-design and digital health, the infrastructure of the program was shaped in such a way that it is now usable on NHS systems and maintains the ethical administration of sensitive information. The collaboration between research teams and Web developers was essential so that the focus of development is not only on the content of the program but also on the user interface and functionality of MyDESMOND.

### Interface Design

We followed a target population-centered approach, based on the taxonomy of approaches to developing DHI [52]. This approach suggests that any intervention must be informed by the actions and views of the people who are end users. Ongoing involvement of the target group and end users is fundamental for successful intervention design. We ensured that people living with T2D and stakeholders were regularly involved throughout the design of MyDESMOND. This co-design process meant that end users were part of an iterative refinement process to certify that the language and content were easy to understand, and to ensure the navigation system was simple to use.

### Theoretical Framework

The role of theories in DHI is crucial, but yet not all programs are guided by theories [53]. It is integral that key theories and models are made explicit in digital programs to enhance understanding of the intended intervention, guide the evaluation process, and inform the implementation of the program in the *real world* [54]. We ensured that MyDESMOND was underpinned by a strong theoretical framework that embedded behavioral theories (HAPA model, Social Support theory, and COM-B model) and the BCT taxonomy to predict and understand behavior change.

### Program Functions

Step 1 of the IM process, which included the mixed methods needs assessment, allowed us to collate findings and insight not only from our own work but also from the current literature. This helped identify a number of key functions that were eventually incorporated in MyDESMOND, including information, personal stories, interactions with other app users, and *buddies*. Literature in chronic self-management has an established series of evidence to confirm that providing a secure platform for communication (ie, in the form of chat forums), allows the end user to share personal experiences, increase their knowledge, and improve self-care [55-57]. In addition, literature around gamification suggests that virtual support from friends and family is associated with increased physical activity and that game design and social incentives (*buddies*) can enhance collaboration, accountability, and peer support [58].

### Future Directions

The evaluation and implementation of MyDESMOND is currently ongoing in the United Kingdom and Australia, and results on its effectiveness will follow [59]. A follow-up paper on evaluation and implementation (step 5 and step 6 of the IM framework) is also currently in working progress. Despite the focus of the program aiming at adults with T2D, the IM framework could help adapt the current digital platform to new populations and health conditions and provide a taxonomy of behavior change that would inform the content and functionality of new digital programs.

### Conclusion

IM framework was an effective approach to bring together research evidence and theoretical concepts to guide the adaptation and development of MyDESMOND. Our evidence- and theory-based digital program provides ongoing support and guidance to people with T2D. We hope that our paper can be used as an example for other researchers and HCPs who are developing similar digital self-management programs across chronic diseases.

## Abbreviations

BCT: behavior change techniques
DHI: digital health intervention
HAPA: Health Action Process Approach
HCP: health care professionals
IM: intervention mapping
NHS: National Health Service
NIHR: National Institute for Health Research
PPI: patient and public involvement
PWA: progressive Web application
SME: subject matter experts
T2D: type 2 diabetes
